# Developing an Extended Model of the Relation between Work Motivation and Health as Affected by the Work Ability as Part of a Corporate Age Management Approach

**DOI:** 10.3390/ijerph15040779

**Published:** 2018-04-17

**Authors:** Annemarie Feißel, Richard Peter, Enno Swart, Stefanie March

**Affiliations:** 1 Institute for Social Medicine and Health Economics, Faculty of Medicine, Otto-von-Guericke University Magdeburg, Leipziger Str. 44, 39120 Magdeburg, Germany; enno.swart@med.ovgu.de (E.S.); stefanie.march@med.ovgu.de (S.M.); 2 Institute of the History, Philosophy and Ethics of Medicine, Ulm University, Parkstraße 11, 89073 Ulm, Germany; richard.peter@uni-ulm.de

**Keywords:** work motivation, health, work ability, corporate age management, lidA-study, older employees

## Abstract

Due to demographic changes, the employee structure in companies is changing dramatically. It will be necessary to offer employees suitable, age-adequate jobs. As one of its foremost goals, optimized business management strategies must create conditions for guaranteeing a person’s health, work ability, and work motivation. In the context of corporate age management concepts, the literature recommends to retain and integrate older employees in the organization. This paper aims at developing an extended model of the relation between work motivation and health as affected by work ability and at deriving a host of measures that enterprises can apply as part of a corporate age management policy to counteract the impact of demographic changes. The model also takes into consideration factors influencing the relation between work motivation and health as affected by work ability (socio-demographic parameters, occupation, work-related stress). Additionally, the extended model translates the literature-based results into a corporate setting by way of a corporate age management program. The model comprises a process focusing on retaining and promoting work ability in order to maintain or boost work motivation and health. The host of measures presented serves as a basis to preventively counter demographic change on an individual, interpersonal, and structural level.

## 1. Introduction

Due to demographic changes, enterprises face dramatic changes in their employee structure. Studies and statistics from the U.S. [1,2], Canada [3], and Europe [4], for example from The Netherlands [5], show a rise in the age of the potential work force and a decrease in the number of employees. Consequently, both society and individual companies perceive an increasing need to offer employees age- and health-adequate jobs in order to retain their productivity in the labor market until they reach legal retirement age (and beyond that, if they so desire) [6]. In addition, the wish to retire early also plays an important role. According to Hasselhorn and Ebener [7], there are persons fit to work who want to quit their professional life for reasons of personal motivation. Various influencing factors such as one’s state of health, work ability, and other important work-related aspects play a central role when a person opts to end his or her career early. The meaning and importance of a person’s job and the challenges connected with it are rather seen as motivators for keeping one’s job, whereas job demands and limited personal freedom or control are considered as risk factors for early retirement [7]. Accordingly, it is difficult to draw a clear dividing line between work motivation and the desire to retire. Early prevention can therefore be seen as a precondition to keeping one’s job until old age. How enterprises will successfully deal with the far-reaching demographic changes in the labor market will have a lasting effect on a company’s success [8].

Success in guaranteeing work participation is largely determined by work ability and work motivation [6]. Therefore, optimized business management strategies must not only aim at protecting health but also at safeguarding the employees’ motivation and work ability [8].

This has consequences for work-related primary prevention [7], which is primarily a company’s responsibility. It should be the one to find adequate solutions. In view of corporate age management concepts, Naegele and Walker [9] recommend to take steps to retain and integrate older people in the labor market. A corporate age management program will enable businesses to respond to and counteract demographic changes, the aging of their staff and the ensuing challenges. A corporate age management policy does not only have advantages for (older) employees but enterprises themselves can and will benefit from it, too [9].

Based on existing models relating both to maintaining and boosting the health of employees as well as motivational aspects, this paper intends to develop an extended model of the association between work motivation and health as affected by the work ability. So far, no previous model has linked age management and theories on health, work ability, and work motivation. In light of demographic challenges and the foreseeable shortage of labor, employees and employers need to consider these aspects within an age management policy. This approach will also create a competitive advantage. In the end, older employees will be able to enjoy better health and be more motivated to work while businesses will benefit from ensuring that older employees continue to work. Therefore, this paper aims at deriving a host of measures that companies can apply to take preventive steps to counteract demographic changes with the help of a corporate age management program.

Initially, this paper will discuss established models focusing on maintaining and boosting employees’ health that also contain motivational aspects. They lay the foundation for developing an extended theoretical model, which this paper centers on. On this basis, we will provide an overview of the latest research findings. Factors having a considerable impact on the relation between work motivation and health will be integrated into the extended model. Finally, insights derived from the literature will be translated into the corporate setting with the help of an age management program.

## 2. Selected Theoretical Models Illustrating the Relation between Work Motivation and Health

The job demand-control model was developed by Robert Karasek in 1979 and is considered to be one of the most important theoretical concepts to explain work-related illnesses [10]. It comprises two dimensions of job-related parameters: (1) Job demands such as time pressure, complexity, and the degree of difficulty and (2) job control in planning and decision-making processes based on autonomy, work-related learning potentials, and skill utilization. The effects of increased demands on the health and wellbeing of employees depend on their degree of control.

In the 1990s, Jeffrey Johnson developed the demand-control model into the demand-control- support model. He expanded the original model by adding the dimension of social support at work [11]. According to his understanding, demanding jobs with low job control (autonomy) and low social support entail a high risk for an employee’s health (isolated high-strain jobs). 

Simultaneously, Johannes Siegrist developed the effort-reward imbalance model [12], which is also referred to as the model of professional gratification crises. This model puts the focus more on rewards and resources and less on control. Rewards are grouped into three categories: financial, socio-emotional (recognition, appreciation), and status-related (career opportunities, job security). In addition, personal coping skills of employees are taken into consideration. According to this model, an imbalance of demands and rewards combined with low personal coping skills considerably steps up the risk of developing a stress-related disorder.

In the past decade, the demands-resources model was developed, which rests on the assumptions of the demand-control model and the effort-reward imbalance model and expands these. In addition to the two dimensions of demands and strains, the model is also based on the assumption that two important psychological processes characterize work. The first path relates to health complaints and implies that chronically high job demands put a strain on the physical and mental resources of employees, which may cause exhaustion and health problems [13]. The second path describes the motivational process saying that the resources of work also function as motivators and result in high job commitment and excellence, thus diminishing adverse health effects [13].

**Figure 1:**
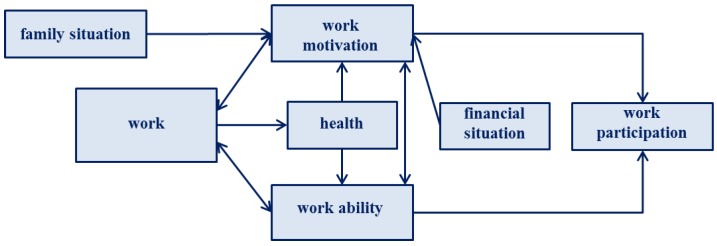
The lidA framework (modified according to Peter & Hasselhorn 2013 [14]).

In 2013 a multi-disciplinary theoretical concept was developed in the context of the “lidA-leben in der Arbeit” study, a cohort study on health, work, age, and work participation [14]. The cohort study examined how various work-related factors influence the health status of older employees [15]. The lidA framework on work, age, health, and work participation illustrates the complexities of work participation of older employees. Figure 1 shows a modified version of the lidA framework from Peter and Hasselhorn. The model rests on the assumption that work participation of older employees affects employment through the determinants of work ability and work motivation. It presents possible effects on work participation and mutual dependencies.

One version of the model contains eleven influencing factors that affect the decision of how long a person will keep working [16]. The work ability and work motivation are determinants of work participation that also effect health. Work ability means being able to work, whereas motivation refers to a person’s readiness and willingness to work [6]. Burr et al. [17] show that health and work participation must not necessarily be mutually dependent: In 2010, more than 50 percent of all people who did not work reported to be in good health while more than 30 percent of the working population rated their state of health as poor. 

## 3. Current State of Research

The association between work motivation and health is theoretically well-established and empirically grounded [18,19,20,21,22,23]. Resulting connections with regard to mental health and psychological disorders such as burnout, depression, or states of exhaustion due to stress at work have been studied and discussed at length [24,25,26,27,28,29]. 

Fernet [30] demonstrates that motivation has an impact on the ability of employees to adjust to their work environment and on their mental health. Salmela-Aro and Nurmi [31] argue that motivation is a key mechanism for maintaining a person’s wellbeing at work. A motivated employee who has a positive attitude to work can handle and avoid stress better. According to Locke and Lathman [24], motivation springs from goals defined by the employees themselves. Progress in reaching one’s goals boosts employee wellbeing and health. In this respect, goals based on an intrinsic motivation, meaning that people feel an inner joy of doing their work, are particularly beneficial to a person’s health [22]. 

Björklund et al. [20] also confirmed the connection between work motivation and subjective wellbeing. They reported about employees with health complaints who were less motivated than those enjoying good health. In their study on changes in work motivation and its effects on health they also found out that the more an employee’s work motivation degrades the higher his or her risk to fall ill. Lohela et al. [21] succeeded in demonstrating that a decline in an employee’s commitment, which is comparable to work motivation, had a negative impact on that person’s health in the long run.

However, work motivation may not only benefit an employee’s mental health but may also increase the risk of mental disorders [21,23,30,31]. According to Salmela-Aro & Nurmi [25] and Pines [25], highly motivated people run the risk of developing burnout. Employees may also suffer from burnout if their working environment does not match their expectations and their job does not offer opportunities for reaching their self-defined goals [26,31]. 

In their longitudinal study examining the association between work motivation and health, Feißel et al. [18,19] identified the work ability as the decisive determinant of work motivation and health. For their analyses, they performed reciprocal directed multifactorial variance analyses with work motivation (Model 1) and health (Model 2), stepwise expanded by work ability and other influencing factors (year of birth, sex, education, income, Blossfeld classification, working hours, and work-related stress). As a result, in model 1, work ability shows its influence on work motivation with constancy also when additional influencing factors were integrated. Model 2 shows work ability as a determinant on health as well as work motivation. Therefore, work ability can be confirmed as an essential determinant of work motivation as well as of health. Consequently, both an employee’s work motivation and subjective state of health increase with his/her degree of work ability [19]. 

Work ability is defined as a person’s capability of coping the demands of work to a given point of time with his human resources [27]. According to Tengland [28], a person has work ability if she has the physical, mental, and social health, manual, intellectual, and social competences, as well as occupational virtues and the relevant job-specific virtues. The higher a person’s competence and health are, and the more flexibility, coping strategies, and occupational virtues she has, the higher her work ability is [28]. Work ability has an effect on an employee’s well-being and influences work disability, sick leave, and early retirement as well as a company’s productivity [29]. Risk factors that threaten older people’s work ability are heavy physical activity, stressful work, and workplaces and a poor organization of work like a lack of understanding, fear of failure, or conflicts [32]. The association between work motivation and work ability is a central aspect of the lidA framework [10]. It refers to the work ability as a factor influencing work motivation. At the same time, motivational factors, values, and attitudes have an impact on a person’s work ability [32]. Illmarinen [33] states that motivation is part of heaving work ability. 

Regarding the association between health and work ability, Ilmarinen [29] confirms that health influences work ability. He postulates health as an individual factor of employees that influences a person’s work ability at a rate of about 40 percent. Nevertheless, it is also possible to have a good work ability despite poor health [29]. So, health is not a necessary requirement for work ability, but people need to have some degree of health, such as being able to walk, concentrate, talk etc. [28]. Also, Tengland [28] states that even if you are perfectly healthy, you might still not have work ability, since most kinds of work require special competences. However, Sun et al. [34] show that low working ability as well as low level of resilience are related to the likelihood of having depression. 

In the light of the empirical results, work ability has a positive effect on work motivation and health. Therefore, it is useful to focus on the work ability of middle-aged employees functioning as a so-called “link” to maintain and enhance the work motivation and health of employees [18,19]. If an employer makes an effort to maintain and promote work ability, this will affect both the employees’ health and work motivation. This means that employers can use the work ability as an adjustment tool to maintain and boost health and work motivation at the same time. This can be considered as a beneficial measure to keep employees engaged at work [19]. Isolated measures to either stimulate work motivation or to boost health seem to be counterproductive. By pursuing an integrated approach, it is possible to save unnecessary (direct and indirect) costs. 

## 4. Factors Influencing Work Motivation and Health

Below, the authors will introduce empirically verified determinants that are seen as highly relevant for introducing and implementing a corporate age management policy. 

### 4.1. Socio-Demographic Parameters

To understand the association between work motivation and health as affected by the work ability, it is crucial to consider the socio-demographic parameters of age and—to a lesser extent—gender. With regard to the work ability, there is consent if age is identified as an influencing factor. According to Prümper and Richenhagen [35], the employees’ work ability generally declines with biological age. They also report that the variability of work ability increases drastically at the age of 45. This is in line with the result of Pojohnen [36], who found that the effect of age on the work ability index (WAI) is significant only after the age of 45. Accordingly, older employees differ more in their work ability than younger people. That is why measures should be tailored to their individual needs. Also, Tuomi et al. [37] show that the probability of a low work ability increases with age. However, Abbashi et al. [38], Wilke et al. [39], and Golubic et al. [40] state no significant impact of age on work ability. These results are in line with Padula et al. [41], who found no differences between work ability in relation to age, but they observed an increase variability of responses for WAI score in older worker. There seems to be no differences between men and women in terms of their work ability [41]. 

Studies of work motivation show no or little differences as regards age and gender. Frerichs [42], Rabl [43], and Koji et al. [44] demonstrate that older employees are not necessarily less motivated than younger ones but that other factors play a role for older and younger employees. In contrast to younger employees, older staff members tend to be more interested in receiving recognition and passing on their knowledge, to have more freedom to make decisions and take control, and to participate in decision-making processes [45]. Feißel [18] reports only slight differences between the genders when it comes to work motivation.

As concerns the subjective state of the health of employees, Feißel et al. [46] and Burr et al. [17] both state that the share of employees with good health declines with age irrespective of their gender. Both age-specific and gender-specific differences have been identified with regard to health complaints. Burr et al. [17] and report an increase in health problems with increasing age that have a negative impact on a person’s subjective state of health. In general, women have more health issues, whereas men often have more serious illnesses such as cancer or cardiovascular diseases which helps to explain why women live longer compared to men [47].

According to the above-cited empirical findings, work motivation is not or hardly dependent on age and gender, however, both socio-demographic parameters have a strong impact on a person’s work ability and/or health. That is why it is essential to take them into consideration in the extended model. In addition, biological age is a factor inevitably tied to an age management strategy. 

### 4.2. Occupation Categories

Belonging to a certain occupation category has been identified as another moderating determinant of the relation between work motivation and health as affected by the work ability. Among others, Hasselhorn and Freude [48], Tuomi et al. [37], and Tuomi et al. [49] studied the relationship between the work ability and current occupation. Hasselhorn and Freude [48] report that the work ability develops in different ways within different occupation categories. For instance, we can expect the work ability of doctors and executives of all age groups to be significantly higher than, for example, that of teachers. The reason for this is the association between work ability and work-related and health-related factors in predominantly mentally challenged occupational groups [48]. Tuomi et al. [37] and Tuomi et al. [49] state that the higher the percentage of heavy physical loading during work is the poorer is the level of work ability.

Feißel et al. [46] demonstrate that employees in higher qualified jobs, so-called white-collar jobs, are both healthier and more highly motivated than employees in low-skilled jobs, so-called blue-collar jobs, who rank among those with the smallest share of employees with good health and high motivation. In their study on motivation and health, Björkelund et al. [20] also confirm that white-collar employees have the highest work motivation among the working population. Presumably, better working conditions and recognition in higher qualified jobs will lead to higher work motivation. In their study, Burr et al. [17] proved the impact of belonging to a certain occupation category on health. They found evidence that employees reporting the largest share of positive health can be found in higher qualified jobs. A number of studies [17,18,20,48] succeeded in demonstrating that a person’s current occupation plays a decisive role for his/her work ability, work motivation, and health. Therefore, it should be factored in as an influencing factor in the model. 

### 4.3. Work-Related Stress

Work-related stress results from an imbalance of demands and control or rewards/recognition at the workplace and has been identified as a factor influencing work motivation, health, and the work ability. Its impact on health and motivation has already been grounded theoretically in the selected models described above [10,12]. That is the reason why work-related stress is applied to the extended model presented. 

According to Schreurs et al. [50], work motivation is negatively associated with job demands and positively with rewards or recognition for work. In the long run, increasing stress has a negative effect on motivation [42]. The relationship between work-related stress and health as well as diseases or health problems has already been studied by du Prel et al. [51], Van Vegchel et al. [52,53], Wilkins and Beaudet [54], as well as Karasek and Theorell [55]. They show that work-related stress has a negative impact on a person’s health and may cause depression, hypertension, and cardiovascular disorders, among others. Feißel [18] also studied how work-related stress affects work motivation and health. They found a trend towards an increase in work motivation and the share of positive health when work-related stress decreased. Inequalities in gratification models and gratification expectations that a person cannot reach in his or her current position cause excessive stress that is harmful to that person’s health which in return may discourage and demotivate an employee [54]. 

**Figure 2:**
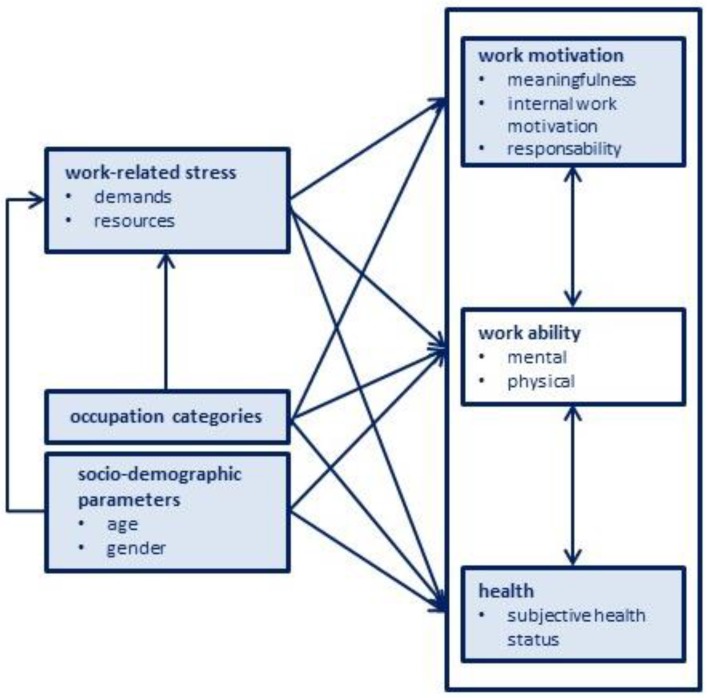
The relation between work motivation and health as affected by the work ability—Expansion to the factors age, gender, occupation categories, and work-related stress.

In summary, the empirical findings show that the socio-demographic parameters of age and gender as well as a person’s occupation belongs to and work-related stress influence the association between work motivation and health as affected by the work ability as a constant factor. Figure 2 illustrates this relationship as a segment of the lidA framework shown in Figure 1, expanded by the influencing factors of age, gender, current occupation, and work-related stress that have been identified.

Finally, we will integrate the above-mentioned models and influencing factors identified into an extended model explaining the association between work motivation and health as affected by the work ability in a corporate setting in view of a corporate age management policy.

**Figure 3:**
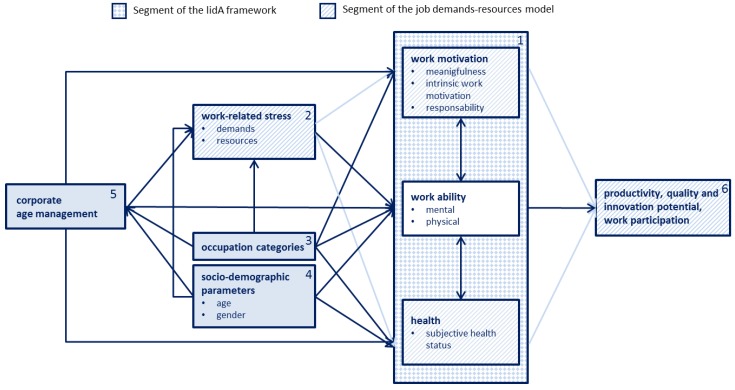
Suggestion for an extended model of the relation between work motivation and health (own diagram).

The model centers on the relation between work motivation and health, both being constantly affected by a person’s work ability. In view of the empirical findings, it makes sense to concentrate on the work ability of middle-aged employees in order to maintain or boost their work motivation and health [6,19]. The three determinants of work participation have already been discussed by Peter and Hasselhorn in their lidA framework [14] (Figure 3, Box 1).

In their job demands-resources model, Demerouti and Bakker [13] refer to work-related demands and resources/rewards that can boost motivation if in balance and may cause health issues due to work-related stress if not in balance. A number of other studies also identified work-related stress to be a decisive factor having an impact on work motivation and health. It has therefore been considered in the model (Figure 3, Box 2).

The type of work or an employee’s occupation category largely determines the content of his or her work and thus demands and resources/strains [56]. It has therefore also been integrated into the model (Figure 3, Box 3). An evaluation of the socio-demographic parameters of age and gender make it possible to identify risk groups having a great need for support (Figure 3, Box 4).

Businesses have an important role to play when it comes to coping with demographic change [9]. Since the perspective on older employees and their value for a company has shifted as a result of demographic challenges and the intended extension of working life [9], a corporate age management policy is gaining importance forming a framework for the theoretical model. In the model, change processes beneficial to an employee’s health and intervention strategies to maintain and promote employee work ability take center stage (Figure 3, Box 5). In an organization, both behavior and condition-oriented measures play a role in this context.

On the one hand, good working conditions and a high quality of life at work promote the employees’ health, work motivation and work ability and help safeguard the work participation of older employees. On the other hand, they raise a company’s productivity, quality, and innovation potential (Figure 3, Box 6) [29].

## 5. Discussion

The model developed in this paper comprises a process focused on maintaining and promoting work ability in order to maintain and boost work motivation and health. According to Burr et al. [17], many people with poor health continue to work. Health issues and illness should therefore not be equated with a poor work ability and retirement. Especially in view of the rise in chronic disorders, this will apply to a growing number of employees in the future [57]. It is possible to maintain their work ability by matching their reduced capacities with their job demands [7].

The solution could be a corporate age management program that helps reduce work-related stress on the one hand and on the other maintains and promotes the work ability as well as work motivation and health. Age management means to integrate the age aspect into all business processes and decisions so that employees can enjoy healthy and motivating work until retirement age [9]. 

According to Sporket [58], for an age management policy to be successful and sustainable participatorily implemented measures have proven to be particularly useful. As a tried and tested instrument of ensuring employee participation as a means to diagnose problems and prepare measures as required, health circles have proven to be successful [59]. Actively participating employees and executives, if required, can discuss issues on an equal footing in order to find solutions agreed on by all people involved. That means solutions are not solely implemented by order of the management (top down) but the people concerned can develop and implement their own grass-root ideas (bottom up) [60]. 

Not only participation but also cooperation and sustainability play a key role when it comes to implementing an age management policy [58]. Companies do not always have the necessary competences to implement special measures so that collaboration with different partners may be beneficial. We recommend cooperating externally mainly with scientific institutions, consulting institutes, or social insurance agencies. Internally, it is useful to cooperate with corporate actors such as the human resources department, the works council/staff representative and/or the occupational safety and health protection officer. Sustainability is a key aspect of an age management policy. Designing work processes to meet age requirements will only spell success if these processes are consolidated in a structure and integrated into everyday work life. It is also possible to integrate measures into existing target agreements or management systems and enshrine them in bargaining agreements and guidelines [58]. 

The authors believe the creation of a parallel structure to be counterproductive. Also, a successful age management policy can only be implemented if it is not only geared to affecting the behavior of individual actors within an organization but if it also creates conditions for an age-adequate organization. Conditions provide clear structures and rules and consequently cause individuals to behave in certain ways. Used positively, an age-adequate organization also offers a chance to behave in age-adequate ways accepted by all people (colleagues and executives). Prevention and health promotion should always comprise three levels: the individual level, the interpersonal level, and the structural level [12].

A good balance of all levels will, on the one hand, increase the employees’ quality of life and wellbeing and, on the other, boost productivity and the quality of work, which consequently promotes business success. The objective is therefore to develop personal competences (behavior) and create age-adequate working conditions (circumstance) that promote health to reach or optimize personal and company goals [35].

Since behavior prevention alone has little effect, it is useful to first focus on preventing certain conditions or circumstances in order to lay the foundations for a healthy and age-adequate work organization [34]. According to Prümper and Richenhagen [35], executives play a key role in this context. Illmarinen and Tempel [61] also showed that leadership has the by far strongest effect on the development of work ability. In practice, it is recommendable to give some thought to how good leadership can work on an individual, interpersonal, and structural level as part of a corporate age management policy. 

The individual level pertains to behavior prevention. Executives can significantly influence their staff’s behavior. They function as role models and essentially shape work culture which forms the decisive basis for health and wellbeing at work. Workplace culture and ethos are significantly related to employees’ health, especially to the likelihood of heaving depression [34]. Demographic change plays a central role in this context. Consequently, it is absolutely necessary to implement an age management program supported, communicated and lived by the company’s management. Better and especially age-adequate leadership has a very positive impact on the work ability of older employees and generates acceptance among “still” young staff members [61].

The interpersonal level aims at improving social relationships at work. Executives are required to communicate to their staff the necessary appreciation, acceptance, and recognition of their performance, to see their potential and to individually maintain and further develop their staff as best as possible in the company’s interest. As Siegrist [12] describes in his effort-reward imbalance model, an imbalance of efforts and recognition or rewards results in a higher risk to fall ill due to stress. Apart from material recognition or rewards such as a pay raise, immaterial aspects in particular, such as timely and well-founded praise for a person’s performance or showing emotions as well as appreciation of a person by showing empathy have been identified. By building friendly and respectful rapport, executives can positively influence the work ability of employees [62]. Large companies can offer non-monetary incentives according to the individual needs of their employees. This mainly pertains to measures improving the work-life balance. These may include the flexible organization of working hours, career opportunities matching a person’s qualifications, giving a job guarantee, and offering in-house services such as corporate sports programs or a corporate kindergarten [60]. Salciuviene et al. [63] discuss the importance of a perceived corporate social responsibility to gain advantages over other companies, to achieve their goals and profits. They develop a theoretical model of small and medium-sized enterprise corporate social responsibility initiative implementation. They show that companies that integrate corporate social responsibility benefit from reduced turnover, increased productivity of their employees and their reputation [63], because corporate social responsibility activities contribute positively to the company’s image [64]. Gharleghi et al. [65] show that corporate social responsibility supports employees creative work involvement as motivational factor, which leads to a positive work attitude and reduces their intention to leave. Shin et al. [66] found that corporate social responsibility is negative associated with counterproductive work behaviors including increased absenteeism and turnover, as well as decreased satisfaction and productivity.

The structural level pertains to a change in working conditions. By expanding autonomy at work, an executive can help maintain his or her staff’s work ability. As described in the demand-control model (see above), high job demands only have an adverse effect on the employees’ health and wellbeing if connected with few opportunities to take control, e.g., limited freedom to make decisions. For business practice, this means it is paramount to expand opportunities for employees to take control to maintain their work ability in the long run, particularly in view of rising work-related stress levels, growing workloads, and the resulting intensification of work [35]. Special measures expanding the scope of control include job enrichment, job enlargement, and job rotation. Job enrichment can be described as a means to extend the responsibilities and autonomy of employees; job enlargement refers to an expansion of tasks without giving an employee more responsibility, and job rotation entails a job change within the company [35]. If used selectively and thoughtfully, these tools may be applied as instruments of age management. In order to offer employees opportunities for learning and growth as well as skill trainings and thus more freedom to take control, it is essential to expand opportunities for in-house and external further trainings. Such trainings play a particularly important role for older employees. They need to use and be challenged to apply their cognitive skills to maintain them or even advance them. But continuous training and learning to strengthen cognitive skills and competences is not only important for older employees but for all staff members. Older employees can use their skills purposefully to train and help younger co-workers, e.g., in mixed teams. That means that executives and corporate culture are challenged to promote lifelong learning as a cross-generational responsibility [29]. If there are employees at risk of losing their earning capacity, early workplace interventions can help to preserve their work ability. Schwarze et al. [67] implemented an intervention on people with musculoskeletal disorders including workplace-management, company medical service and work-related out-patient medical prevention and rehabilitation. As a result, they show an improved work ability, health status, and working capacity and a reduction of time lost due to sick leave.

Depending on the measures implemented, executives have a double function when it comes to maintaining and promoting their staff’s work ability. On the one hand, they are role models for age-adequate, healthy behavior and must lead in age-adequate and healthy ways; on the other hand, they must communicate decisions and measures planned as part of a health-oriented management strategy applied to the individual levels described before. The following Table 1 illustrates once again the three levels using the examples mentioned above, supplemented with other measures that might be applied within an age management program.

## 6. Conclusions

For securing work participation, optimized business management strategies must not only aim at protecting health but also at safeguarding the employees’ motivation and work ability.

This can be done as part of a corporate age management program, which will enable businesses to respond to and counteract demographic changes, the aging of their staff, and the ensuing challenges. The developed extended model of the association between work motivation and health as affected by work ability linked age management and theories on health, work ability, and work motivation. Older employees will be able to enjoy better health and be more motivated to work while businesses will benefit from ensuring that older employees continue to work. The measures help companies to apply to take preventive steps to counteract demographic changes with the help of a corporate age management program.

## Figures and Tables

**Table 1:** Concrete measures that may be applied as part of an age management program to maintain and strengthen work ability.

Level	Measures
individual	being a role-model behavior-based preventive approach to overall working conditions (environment promoting physical activities, healthy nutrition at work, addiction prevention, stress management, etc.)
interpersonal	appreciation/acceptance of the experience/competences of older employees participation, participation in decision-making, involvement, empowerment creating transparency, meaningfulness, offering support integration in personnel development and recruiting processes flat hierarchies culture of trust age-adequate staff orientation mixed teams corporate social responsibility activities
structural	extending opportunities for control/autonomy offering further training opportunities need-based working time and vacation planning reduction of job demands integration into existing management systems age-adequate design of workplaces, work organization and work environment incentive systems for remaining with the company
